# Decay of Competence with Extended Research Absences During Residency Training: A Scoping Review

**DOI:** 10.7759/cureus.5971

**Published:** 2019-10-22

**Authors:** Nada Gawad, Molly Allen, Amanda Fowler

**Affiliations:** 1 Surgery, University of Ottawa, Ottawa, CAN; 2 Emergency Medicine, University of Toronto, Toronto, CAN; 3 Surgery, Memorial University of Newfoundland, St. John's, CAN

**Keywords:** residency training, research training, research leave, academic leave, competence decay, skill decay

## Abstract

A significant number of residents in postgraduate training programs pursue dedicated research training. Currently, no formal curricula exist to transition residents back into clinical roles following dedicated research leave. This scoping review aims to determine what literature exists on the challenges faced by trainees who interrupt their clinical training for extended periods of time for research leave. The Pubmed and Medline databases were searched for all study designs related to postgraduate trainees taking academic or research leave. A three-step selection process including title, abstract and full-article review was employed to identify articles that mentioned decay of knowledge, skill or competence. A narrative review of the literature was generated to present key themes identified within the studies. The search yielded 174 articles of which five investigated resident skill decay during research leave. The five studies included for analysis were cohort studies that used general surgery residents’ self-perception and faculty members’ perception of residents’ skill decay as a measure. Residents and faculty perceived decay of residents’ technical skills, leadership skills and knowledge following dedicated research leave. The greatest decay perceived was in technical skills, specifically with more complex tasks and longer periods of non-use. This review identified that residents and faculty perceive a decay of resident skills following dedicated research training. To provide the necessary support to limit this potential decay, as well as to assist in the transition back into clinical training, the needs of and challenges faced by research residents and postgraduate programs must be better understood.

## Introduction and background

Research training influences the trainees’ future career and academic activities [1-5]. It is associated with better clinical care and is instrumental in academic teaching environments [6]. The CanMEDS roles of The Royal College of Physicians and Surgeons of Canada recognize the importance of research training by including scholarship as one of the core competencies for physicians [7]. Similar competencies are also put forth by other international licensing bodies [8-9]. The Clinician Investigator Program (CIP) is an RCPSC-accredited program that aims to assist residents in becoming clinician investigators [10]. The program involves a minimum of two years of mentored research-intensive training with enrollment in a graduate degree or postgraduate stream to complete a thesis [10]. Research residents continue to engage in clinical responsibilities (including on-call experiences) and academic responsibilities throughout dedicated research training, albeit limited and to varying degrees. Fifteen Canadian universities have accredited programs with approximately 200 residents participating at any given time. When the trainees are enrolled in surgical residency programs, their participation in CIP is often synonymously called the Surgeon Scientist Program (SSP) [11-13]. The number of Canadian postgraduate trainees enrolled in Clinician Investigator programs, including CIP, MD/PhD, and MD/MSc programs, has demonstrated significant growth over the past several years [14]. The concept of research years mid-residency is not unique to Canada, as similar programs known as Clinician Investigator Training Programs (CITP), exist at institutions in the United States [15-16]. Currently, the transition back from SSP/CIP into clinical training involves returning to residency as though no interruption occurred. While some informal re-introduction to clinical duties exists, to the best of our knowledge, no formal or standardized process exists to assist residents in reintegrating into their clinical responsibilities. Furthermore, while some institutions require that residents resume training where they left off, other institutions promote residents by a full clinical year despite having spent one or more years engaged in research with limited and variable clinical training. In the contemporary setting of Competency Based Medical Education (CBME), this promotion model represents a time-based educational approach, which contradicts the notion of tailoring learning to trainees’ abilities and needs [17]. There is considerable literature on the decay of knowledge and technical skills over time yet when this time is devoted to SSP/CIP training, no formal curricula exist to transition residents back into their clinical roles after formal research leave [18-26]. The purpose of this scoping review is to determine what literature exists on the challenges faced by trainees who interrupt their clinical training for extended periods of time for research leave. Such trainees are referred to as research residents throughout the remainder of the text. For the purpose of this scoping review, research leave is defined as a minimum of six months of research-intensive training with enrolment in a graduate degree, postgraduate stream or fellowship, and which involves a significant decrease in clinical responsibilities. Our aim is to review the literature to explore the effects of such interruptions on residents’ clinical training and overall clinical competence. In this context, the potential implications of knowledge and skill decay are discussed from the perspectives of patient safety, resident well-being, and training-program needs with the intention of improving our understanding of the gaps and deficiencies in transitioning residents directly back into clinical training without formal support mechanisms. 

## Review

Methods

An initial search of the literature demonstrated a paucity of relevant literature on the challenges faced by residents who pursue research training; therefore, a scoping review was employed for the purpose of both examining the extent of the available literature and identifying gaps in the existing literature [27]. It is known that trainees experience skill decay following periods of non-use [18,20-21,24-26]. Yet in practice, no curricula exist to transition research residents, who experience varying levels of non-use depending on clinical and academic responsibilities, back into clinical training [15,28]. Given the number of residents participating annually in such leave across all specialties, a greater understanding of the effects of research leave is a critical first step to inform future curricular development [14]. Thus, this scoping review sought to determine whether the medical education literature had studied the impact of research leave on stakeholders, including trainees, patients and training programs. This scoping review was based on the Arksey and O’Malley’s methodological framework (Steps 1-5) for conducting a scoping review: 1) identifying the research question; 2) identifying relevant studies; 3) study selection; 4) charting the data; and 5) collating, summarizing and reporting the results [27]. *Identifying the Research Question*

The research question was identified as: what are the challenges faced by trainees who interrupt their clinical training for extended periods of time for research leave? The research question was kept broad in an attempt to encompass all postgraduate training programs. This was based on an initial review of the literature which revealed a paucity of literature on skill decay following research leave. *Identifying Relevant Studies*

Next, relevant studies were identified through a detailed search strategy with the assistance of a university-affiliated librarian. The electronic databases PubMed and Medline were searched for publications up to and including April 2019. All study designs were included. Iterative modifications were applied to the search strategy based on the citations generated. During the initial search, it was discovered that research leave during residency training is referred to by a variety of terms, most commonly SSP and CIP, and thus variations of both terms were included in the search strategy (Table 1). Furthermore, it was noted that the term academic leave was used interchangeably to describe research leave and was therefore included in the search strategy. Non-English language material was excluded. Articles including some variation of the terms SSP or CIP as well as the reference to knowledge, skill, or competence were included to broadly capture literature discussing any deterioration of those listed. The detailed search strategy is outlined in Table 1. Next, grey (non-peer reviewed) literature was explored using a variety of Internet search engines. A combination of the search terms outlined in Table 1 was used to search non-peer reviewed literature. Finally, to identify additional potential sources and articles, the reference lists of key papers identified through the database and Internet searches were “hand-searched” by two authors (N.G. and A.F.). The methodological quality of individual studies was not quantified, as a scoping review is not designed to weigh the quality of studies but rather intended to be a sensitivity literature search [27].

**Table 1:** Literature search strategy .tw.: text words; .ti: title

Search #	Search Term	Results
1	"Internship and Residency"/ or "Fellowships and Scholarships"/	47230
2	(resident* or residency or fellow*).tw.	179933
3	1 or 2	199180
4	8 Research Personnel/ed [Education]	1911
5	clinical investigator*.tw.	1389
6	(Clinic* Investigat* adj2 (program* or educat*)).tw.	74
7	clinical scientist*.tw.	509
8	((surg* or physician*) adj scientist*).tw.	872
9	Research/ed or research.ti.	214231
10	Biomedical Research/ed [Education]	1350
11	or/4-10	218266
12	3 and 11	3656
13	Clinical Competence/	80742
14	((skill* or competenc* or abilit* or knowledge) and (decay or degrad* or loss or lose or fade or decline or retention or retain*)).tw.	142499
15	13 or 14	221700
16	12 and 15	168
17	limit 16 to English language	154
18	remove duplicates from 17	150

Study Selection

Two authors (N.G. and A.F) independently screened all titles and abstracts to determine which articles addressed the research aim. Any study deemed relevant by either author during the title and abstract screen was included for full-text review so as to capture as many studies as possible and avoid excluding relevant studies at this stage. Each article selected for full-text review was read by both authors. The same two authors then independently applied the inclusion and exclusion criteria developed post hoc, in keeping with Arksey & O’Malley’s methodological framework, to all selected citations during a full-text review to determine which citations to include for final analysis. Studies included for the final analysis were as follows: 1) related to postgraduate trainees taking academic or research leave and 2) mentioned decay of knowledge, skill or competence. Copies of the full article were obtained for those that fit with the research aim. *Charting the Data*

Data were extracted from the studies included for final analysis and charted independently by all authors in keeping with a descriptive analysis approach to data extraction [27]. Extracted data included demographic data (authors, year, study location, study type), methodology (objectives, study design), key findings relating to skill, knowledge, or competence decay, and subsequent themes relating to skill knowledge, or competence decay. Individual study data extraction was discussed between authors, and categories modified iteratively to develop a common language of themes that were identified. This is discussed further below. *Collating, Summarizing, and Reporting the Results*

From the charted data, a narrative review was then generated, both as a table presenting study characteristics, and as a presentation of key themes identified within these studies. This process involved a qualitative review of the key findings relating to skill, knowledge, or competence decay from each article and categorization of these findings into themes. Themes were not established prior to analyzing the data, but rather the key findings were summarized by the authors individually during data extraction and themes regarding decay of skill, knowledge, and competence emerged from these key findings. Themes were modified iteratively during this process. 

Results

Study Characteristics

The initial search strategy applied to Pubmed and Medline electronic databases yielded 174 articles. No literature was identified through the grey literature and hand-search. After applying the three-step selection process including title, abstract, and full article review, only five articles investigating resident skill decay during research leave were identified and included in the final analysis. All five articles pertained to surgical training programs. No literature was identified in other specialties. No discrepancies between authors about study inclusion at the title and abstract stage were excluded. Given the paucity of relevant literature, there was no disagreement between authors about study inclusion for final analysis. All articles were written in the last four years and pertained to general surgery residents. Four of these articles were written by the same research group at the University of Wisconsin School of Medicine and Public Health in the United States [29-32]. The remaining article was written by a research group at UC Davis School of Medicine in the United States [33]. All five studies were cohort studies, and all used general surgery residents’ self-perception of skill decay as a measure. One study also included general surgery faculty members’ perception of residents’ skill decay [32]. Measured skills included technical, knowledge and leadership skills (Table 2) [29-33].

**Table 2:** Summary of articles included in scoping review of decay of competence in research residents ^a^All articles discussed decay of one or more area

Study Characteristic	Number of studies (%)
Study Type	
Qualitative	0 (0)
Quantitative	5 (100)
Mixed Methods	0 (0)
Opinion	0 (0)
Study Location	
North America	5 (100)
Canada	0 (0)
United States	5 (100)
Europe	0 (0)
Other	0 (0)
Year	
<2000	0 (0)
2000-2010	0 (0)
>2010	5 (100)
Research leave associated with the decay of:	
Technical skills	5 (100)^a^
Skills in relation to skill difficulty	2 (40)^a^
Leadership skills in relation to technical skills	1 (20)^a^
Skills in relation to time and exposure	2 (40)^a^
Knowledge in relation to procedural steps	3 (60)^a^

Technical Skills Decay

D’Angelo et al. (2015) aimed to determine if general surgery residents would perceive a greater reduction in clinical skills during research time, especially for procedures they were less confident performing prior to their dedicated research leave [29]. In this multi-center study, residents (*n* = 38) who had completed an average of nine months of research leave completed surveys before and after participating in four simulation tasks (i.e. urinary catheterization, subclavian central line insertion, bowel anastomosis, and laparoscopic ventral hernia repair). This process was used to determine: 1) perceived reduction in global clinical/surgical skills and procedure-specific performance and 2) confidence in perceived difficulty of the surgical tasks before and after performing them. The greatest perceived reduction by residents in global clinical skills was specifically in technical skills (*p* < 0.001; Table 3). 

**Table 3:** Themes of research resident skill decay of five articles discovered in scoping review [29-33]

Theme (Frequency)	Methods (Citation)	Key Findings
Technical skills decay (n = 5; 100%)	Survey of residents’ perceived skill reduction performing simulation tasks (D’Angelo et al., 2015)	Greatest perceived reduction was in technical skills
	Survey of residents’ perceived skill reduction after one-year research leave compared to a new cohort of residents (Jones et al., 2016)	Both cohorts report decay in technical skills among the greatest reduction
	Comparison of residents’ perceptions of technical skill decay to observed performance of leadership skills (Gannon et al., 2016)	Residents reported decay of technical skills during dedicated research leave
	Comparison of faculty members’ perception of residents’ skill decay to residents’ perceptions (D’Angelo et al., 2018)	Faculty perceived residents in dedicated research fellowships demonstrate less technical skill and require more instruction
		Faculty and residents reported the largest perceived skill decay in technical skills
	Survey of residents’ perceived clinical competency in comparison to skills before dedicated research leave (Grova et al., 2017)	Majority of residents perceived a decline in technical skills following the dedicated research leave
Skill decay in relation to skill difficulty (n = 2; 40%)	Survey of residents’ perceived skill reduction and confidence performing simulation tasks as correlated with perceived difficulty of the task (D’Angelo et al., 2015)	Greatest perceived decay in more complex procedure-specific skills compared to simpler procedures
	Comparison of returning and a new cohort of residents’ perceived skill reduction as correlated with a perceived difficulty of the task (Jones et al., 2016)	Greatest perceived skill decay related to tasks requiring higher levels of decision-making, problem-solving, and technical skill for both cohorts of residents
Leadership skill decay in relation to technical skills (n = 1; 20%)	Assessment of residents’ leadership skills defined as the number of directional instructions given to assistant compared to the survey of perceived self-efficacy (Gannon et al., 2016)	Lower self-reported decay in intraoperative decision-making and technical skill correlated with greater number of instructions given to assistant
		Greater number of instructions correlated with tasks perceived to be less difficult
Skill decay in relation to time and exposure (n = 3; 60%)	Survey of residents’ perceived skill reduction and confidence as correlated with the number of months engaged in research and participation in on-call procedures (D’Angelo et al., 2015)	Level of perceived reduction in technical skills and the knowledge of procedure steps correlated with the number of months engaged in research
		Residents who reported engaging in procedures on-call had greater perceived confidence for
	Survey of perceived skill reduction of research residents after returning to clinical training compared to a new cohort of research residents (Jones et al., 2016)	Expected level of technical skill decay correlated with the number of months engaged in research
	Survey of residents’ perceived clinical competency in relation to the type of research completed and number of years dedicated to research (Grova et al., 2017)	Basic science residents spent longer periods in research and perceived greater decay in clinical judgment and patient care skills compared to other forms of research leave
		Residents who spent >two years in research leave were more likely to perceive a decline in aptitude, technical skills, and patient care skills
Knowledge decay in relation to procedural steps (n = 3; 60%)	Survey of residents’ perceived knowledge decay related to procedural steps while performing simulation tasks (D’Angelo et al., 2015)	Second greatest perceived skill reduction in the knowledge of procedure steps
	Survey of residents’ perceived skill reduction related to knowledge of procedure steps (Jones et al., 2016)	Both returning and new cohort of research residents report decay in the knowledge of procedure steps
	Survey of faculty members’ perception of residents’ skill decay in comparison to residents’ self-perceptions (D’Angelo et al., 2018)	Residents’ report the second greatest perceived decay in the knowledge of procedure steps
		Faculty perceived decay of knowledge of procedure steps as the third greatest reduction

A follow-up study to D’Angelo et al.’s (2015) multi-centre study by Jones et al. (2016) set out to assess the same tasks (urinary catheterization, subclavian central line insertion, bowel anastomosis, and laparoscopic ventral hernia repair) performed by general surgery research residents (*n* = 46) in a longitudinal fashion and to determine if perceptions of skill decay among returning research residents (*n* = 16) change over time with reassessment [31]. In addition, the perceptions of the returning research residents were also compared to a new cohort of research residents (*n* = 30) being assessed for the first time. Returning research residents had completed on average 14.2 months of dedicated research training while new residents had completed 6.54 months. Both cohorts of residents in this study perceived a reduction in technical skills. Specifically, returning residents perceived a reduction in technical skills as the second greatest reduction in global clinical skills following knowledge of procedure steps, whereas returning residents rated technical skills as the greatest skill reduction (*p *< 0.05; Table 3). As part of a larger longitudinal study by the same research group at the University of Wisconsin, Gannon et al. (2016) compared self-perceptions of general surgery research residents’ (*n* = 36) technical and non-technical skill decay to observed performance of leadership skills through use of an operative assistant during a simulated bowel repair [30]. On average, residents had spent eight months in dedicated research leave. A self-efficacy questionnaire administered to residents demonstrated self-reported decay of technical skills during dedicated research training (Table 3). In the most recent of the four studies by this research group at the University of Wisconsin, D’Angelo et al. (2018) expand on data from their longitudinal study in 2014 and 2015 [29,31-32]. For the first time, general surgery faculty members (*n *= 77) were evaluated on their perception of general surgery residents’ (*n *= 97) skill decay for those in dedicated research fellowships and to compare faculty perceptions with research residents’ self-perceptions. The majority of faculty agreed that research residents demonstrated less technical skills (63%), and both faculty and residents report the greatest decay of global clinical skills in technical skills (*p *< 0.001) specifically (Table 3). Similar to the studies described above, Grova et al. (2017) assessed former and current general surgery residents (*n *= 19) who had completed at least one year of dedicated research training on their perception of their clinical competency upon return to clinical training [33]. The majority of residents (63%) perceived an overall decline in their technical skills upon return to clinical training from dedicated research leave (Table 3). *Skill Reduction Related to Skill Difficulty*

The level of perceived skill reduction during research leave has been shown in our review to relate to the level of difficulty of the skill. In addition to showing the greatest perceived reduction in technical skills specifically, D’Angelo et al. (2015) demonstrated that residents perceived the greatest reduction in those procedure-specific skills that they perceived to be more complex (i.e. bowel anastomosis and laparoscopic ventral hernia repair) when compared to those perceived as being simpler procedures (i.e. urinary catheterization, central line insertion; *p *< 0.001) [29]. This was again demonstrated in the follow-up study by Jones et al. (2016) in which both the returning and the new cohorts of research residents perceived the greatest reduction of skill in tasks that require higher levels of decision-making, problem-solving, and technical skill (termed open-loop tasks, such as bowel anastomosis and laparoscopic ventral hernia repair; Table 3) [31]. *Leadership Skills Related to Technical Skills*

In Gannon et al.’s (2016) study, dialogue between a resident and an operative assistant was coded to identify the number of directional instructions provided during the simulated bovine bowel repair [30]. This was compared to measures of self-efficacy via a questionnaire. The findings of this study demonstrated that lower levels of self-reported decay in intraoperative decision-making and technical skill correlated with a greater number of instructions given to the assistant (*p *= 0.017). Thus, perceived technical skill decay during research leave was related to assessment of leadership skills. A higher number of instructions given to the assistant also correlated with lower perceived difficulty of the task (*p *< 0.016; Table 3). *Time- and Exposure-related Decay* In D’Angelo et al.'s (2015) initial study demonstrating a perceived reduction in technical skills, the level of the self-reported perceived reduction in surgical skills (*p *= 0.007) and knowledge of procedure steps (*p *= 0.015) correlated directly with the number of months residents had participated in research [29]. This premise was further supported in Jones et al. (2016) and Grova et al.'s (2017) data [31,33]. In comparing the cohorts of new residents versus those being reassessed, new residents had completed an average of 7.66 months less research than those returning residents being reassessed. More time away from clinical training for dedicated research yielded increased concerns regarding skill decay (*p *= 0.023) [31]. Similarly, residents in basic science research who spent more time in dedicated research leave compared to those in clinical, education or other forms of research, perceived a greater decay in their overall aptitude (*p *= 0.061), clinical judgment (*p *= 0.013), and patient care skills (*p *= 0.002; Table 3) [33]. In addition to the time spent in dedicated research, exposure to technical skills during research years emerged as an element pertaining to perceived skill decay. Residents in the D’Angelo et al. (2015) study were more likely to have higher pre- (*p *= 0.004 and *p *= 0.012) and post-procedure (*p *= 0.002 and *p *= 0.041) confidence in their skills during more difficult procedures (bowel anastomosis and laparoscopic ventral hernia repair respectively) if they reported engaging in surgical procedures on call when compared to those residents whose on-call experiences were limited to bedside care (Table 3) [29]. *Knowledge Decay in Relation to Procedural Steps*

Three of the five studies, which used simulated-based procedures for assessment, also noted a reduction in general surgery research residents’ self-perceived knowledge of procedure steps during research leave [29,31-32]. In D’Angelo et al.’s (2018) study, faculty members also perceived a reduction in knowledge of procedure steps (*p *< 0.001) among residents returning from dedicated research fellowships [32]. Interestingly, Jones et al. (2016) demonstrated that in a cohort of new residents beginning dedicated research leave, the knowledge of procedure steps was rated having the most decay followed by technical skills, whereas residents returning after one year of dedicated research leave reported decay in knowledge of procedure steps second to decay of technical skills (*p *< 0.05; Table 3) [31].

Discussion

Our scoping review yielded only five studies specific to competence decay in research residents, all of which were limited to general surgery residents and perception-based studies [29-33]. Albeit limited, the data demonstrates that residents perceive competence decay following research leave. The greatest decay perceived was in overall technical skills, specifically with more complex procedural tasks and longer periods of non-use. Residents also perceived a decay in leadership skills while performing technical skills. 

However, a review of the wider literature yielded a considerable number of studies on the decay of knowledge and technical skill over time, with some literature on mitigating this decay during periods of non-use [18-21,23-26,34-37]. General surgery residents who underwent laparoscopic surgery training were found to have decreased performance six months following initial proficiency training [18,21]. Similarly, gynecology trainees experienced laparoscopic skill deterioration between six and eighteen months of non-use [20]. Gastroenterology fellows who underwent endoscopy training with intermittent breaks were found to have a decrement in endoscopy abilities, with an association between the length of the break and skill decay [24]. Fourth-year medical students who were trained to proficiency in bronchoscopy training experienced skill decay at two months without practice [25]. Nephrology fellows performing hemodialysis catheter insertions were found to have statistically significant skill decay at one year following periods of non-use [26]. Furthermore, military literature on surgical skills suggests that “after 365 days of non-use or non-practice, the average participant’s performance was reduced by almost a full standard deviation (d = -0.92)” [22]. Beyond medicine, the literature on the decay of flying skills among pilots suggests that “no one comes back after a hiatus of any duration (more than a few months) at greater than 80% proficiency…[and] for long hiatus, proficiency upon return is estimated at 40-30%” [38]. Although research residents may experience skill decay similar to the participants in the broader literature described above, several important distinctions may limit the generalizability of these findings to research residents. Specifically, research residents often must demonstrate above-average clinical performance to be considered for research leave, may have higher than average motivation to maintain clinical skills, and may be exposed to varying levels of clinical activities during their research leave [15,28]. However, the potential applicability of the well-established decay of competence over periods of non-exposure in the existing literature underscores the importance of studying and understanding the decay of competence among research residents, as well as using it to inform and develop strategies to transition research residents back to clinical practice. Given the existing wider literature on knowledge and skill decay, the effects of time away from clinical training on patient safety must be considered. Procedural complications are fewer when residents are trained to proficiency, thus, the concern for patient safety given the long hiatus from clinical training when residents pursue research is legitimate [39]. Patient care may also be expected to be impacted by resident well-being, with mental well-being among residents shown to be associated with enhanced empathy [40]. The implications of knowledge and skill decay on resident well-being upon their return to clinical training is an important perspective. Depression and anxiety have been described by first-year trainees facing the transition from medical school to postgraduate residency training, characterized in the grey literature as “a sense of helplessness that develops when one is constantly criticized …[and] made to feel incompetent by supervisors” [41]. It is not hard to imagine how this might parallel the experience of returning research residents, who are experiencing perceptions of skill decay in part due to their dedicated research training, and lower levels of confidence upon the return to clinical training [29-33]. In addition, the change in lifestyle from a more flexible schedule while engaging in research to that of the rigorous and demanding grind of residency may affect resident well-being [42]. More specific to surgical residents, the vicious cycle of insufficient operative volume that currently plagues many junior residents may be perpetuated by throwing research residents back into clinical training unprepared due to the “trickle down” effect of knowledge and skill decay [43-44]. Inadequate technical skill exposure during research training may compound this effect [29]. The downstream effect this has on trainees who are junior to research residents may be a concern faced by junior residents and residency programs alike. For example, a senior resident who has not interrupted their clinical training is often able to delegate parts or all of a less complex procedure to their junior colleague. However, skill decay in a resident returning from research training results in a need for extra practice, and thus the returning resident is unable to afford their junior colleague the same opportunity as when working with a technically stronger resident [30]. Not only might this create a culture of resentment toward working with research residents, but it may also delay the skill acquisition of junior learners if a program has a significant number of residents returning from research. Those who have had inadequate procedural exposure leading to skill decay will perpetuate the cycle of further decreased skill exposure. Thus, when the junior resident with less experience becomes a senior themselves, he or she may not feel confident enough in their technical skills to delegate operative tasks [29,31-32]. This in turn may reflect poorly on resident satisfaction and reputation of a program among new applicants. To apply the findings of the described literature to research residents, they must be interpreted within the context of a surgical residency program. Despite the broad scope of our search strategy, the available data identified has only been studied in general surgery, largely focusing on the decay of technical skills, and therefore may be less relevant to those disciplines that require less technical skill than a surgical resident. In regard to surgical residency training programs, most residents currently pursue their research training after their PGY2 year. Given there is less decay for greater levels of pre-existing proficiency, one might be justified in suggesting residents instead pursue their research training in later stages of training [19-20]. The current time-based structure of surgical residency, however, provides a natural interruption point after PGY2 when residents transition from "junior" to "senior", with the focus of their learning often shifting from ward management to operative management. Because of this shift, research pursuits in later stages of training may instead generate greater interruption to their technical skill learning curve, contrary to the findings of these studies. However, the benefit of pre-existing proficiency may be better applied to the selection of research residents by underscoring the importance of selecting technically strong residents. With significant literature describing the decay of knowledge and skill after long hiatuses across many professions including aviation, military, and medicine, the application of these findings to research residents is certainly thought-provoking. One important distinction, however, is that unlike absences for maternity or sick leave, the majority of research residents still engage in call shifts in an attempt to alleviate decay. However, case exposure, level of responsibility, and frequency of call shifts are variable, and the time dedicated to these clinical responsibilities is significantly decreased in comparison to non-research residents. The optimal exposure to clinical training for skill retention has been demonstrated to be important but has yet to be determined and would be an important area of study as highlighted by this review [23]. Furthermore, whether exposure can be provided in the simulation setting instead has not been studied, although extensive literature on the transferability of simulation training for surgical skills and airway management suggests simulation may be an alternative or supplemental source of exposure [45-48]. This scoping review identified a key gap in the current literature. The available literature represents the perspectives of faculty and trainees on residents’ skill decay following research leave but has yet to objectively measure whether perceived decay equates to objective decay and what the consequences may be. As emphasized by this review, there is a need for objective ways to measure decay, which represents an important area for future research. Furthermore, there remains an opportunity for further research on skill decay following research leave, in particular in non-surgical training programs, given the existing literature is limited to general surgery residents. If decay truly exists, it would likely be of benefit to all stakeholders, including trainees, patients, and training programs to provide a framework to mitigate decay of skill in research residents. It is suggested that frequent, short, distributed training sessions throughout a resident’s research years may be of benefit [18]. Based on the described literature, these training sessions would be best focused more so on open-loop tasks (such as those involving problem-solving as opposed to tasks with discrete responses), accuracy-based tasks, and cognitive tasks as these are more prone to decay [22,31]. The self-assessment and expectations of residents as they progress through research time may pinpoint vulnerable skills in need of re-training upon return to clinical duties. Finally, in addition to medical knowledge and technical skill, many other competencies are expected of residents. The decay of these other vitally important roles, such as communication, leadership, and teamwork remain understudied. 

## Conclusions

Although there is a paucity of literature, and all of which are perception-based studies, this scoping review has demonstrated that surgical residents perceive decay of competence as a result of dedicated research training. The greatest decay perceived is with respect to technical skills, specifically with more complex tasks and longer periods of non-use. To provide the necessary support to limit the decay of a broad range of abilities required of residents, as well as to assist in the transition back to clinical training, the needs of and challenges faced by research residents must be better understood. While the available evidence substantiates that research residents are vulnerable to skill decay, there is no available literature objectively measuring skill decay or describing strategies to support residents as they transition back to clinical duties. One potential solution is to provide exposure to clinical training through simulation, with distributed short training sessions to help mitigate skill decay throughout research training. Further investigation into the challenges of trainees undertaking research leave and that of their postgraduate programmes will be an important next step in the effort to design a curriculum that ensures that these trainees, as well as their patients and colleagues, are not disadvantaged because of their academic pursuits. 
